# A longitudinal epidemiological study on the triglyceride and glucose index and the incident nonalcoholic fatty liver disease

**DOI:** 10.1186/s12944-018-0913-3

**Published:** 2018-11-20

**Authors:** Rongjiong Zheng, Zhennan Du, Mingming Wang, Yushan Mao, Wenjie Mao

**Affiliations:** 1 Department of Pulmonology, Ningbo Yinzhou Second Hospital, Ningbo 1 Qianhe Road, Ningbo, 315192 China; 2 Department of Endocrinology, The Affiliated Hospital of Ningbo University School of Medicine, 247 Renmin Road, Ningbo, 315020 China

**Keywords:** Nonalcoholic fatty liver disease, The triglyceride and glucose index, Insulin resistance, Epidemiology

## Abstract

**Background:**

Triglyceride and glucose (TyG) index and nonalcoholic fatty liver disease (NAFLD) both bave been related to insulin resistance (IR). The study aimed to investigate the longitudinal relationship between TyG index and NAFLD and to evaluate the ability of TyG, through comparing with the predictive value of other indexes, to identify individuals at risk for NAFLD.

**Methods:**

Four thousand and five hundred thirty nine subjects without NAFLD initially were followed up for 9 years. Cox regression models were used to analyze the risk factors of NAFLD.

**Results:**

Cox regression analyses indicated the TyG index was independently and positively associated with the risk of incident NAFLD. In receiver operating characteristic (ROC) curve analysis, the optimal cut-off level for TyG to predict incident NAFLD was 8.52 and the area under the ROC curve (AUC) was 0.76 (95% CI 0.74–0.77), which was larger than that of TG, ALT and FPG.

**Conclusion:**

This study demonstrated that the elevation of the TyG index might predict increase risk for incident NAFLD and it may be suitable as a diagnostic criterion for NAFLD.

## Background

In recent decades, nonalcoholic fatty liver disease (NAFLD), including nonalcoholic fatty liver, nonalcoholic steatohepatitis, hepatic fibrosis, cirrhosis, and hepatocellular carcinoma [1], has been paid close attention and become a major global public health issue [2]. It is estimated that the prevalence of NAFLD was about 20–30% among the world’s population in 2013 [3]. Also, the overall prevalence of NAFLD in mainland of China was about 20.09% (17.95–22.31%) [4]. As a multi-systemic metabolic disease, NAFLD is closely related to many chronic diseases, including metabolic syndrome (MS), type 2 diabetes mellitus (DM), obesity, dyslipidemia and cardiovascular disease (CVD) [5–9], dramatically increasing the clinical and economic burden [10]. Moreover, NAFLD can no longer be considered a mere hepatic manifestation of the MS; increasing evidences suggest that NAFLD can anticipate the development of MS and DM [11, 12]. Considering the seriousness of the disease, early identification for the risk of NAFLD is important and an effective diagnostic predictor for the incident NAFLD should be explored nowadays.

Additionally, recent investigations revealed that NAFLD was closely related to insulin resistance (IR) and many endocrine derangements [13, 14], and the triglyceride and glucose (TyG) index also has been related to IR [15]. However, no consistent conclusions about the TyG index and incident NAFLD have been reached at present. Therefore, we collected 9-year longitudinal follow-up data in order to investigate the longitudinal relationship between TyG index and NAFLD, and to evaluate the ability of TyG index, through comparing with the predictive value of triglyceride (TG), alanine aminotransferase (ALT), fasting plasma glucose (FPG), to identify individuals at risk for incident NAFLD.

## Materials and methods

### Study population

The study subjects, who were the petrochemical employees from Zhenhai Lianhua Hospital, were enrolled in 2006 and were followed-up until 2015 in Ningbo, China. The annual physical health examinations in our cohort study were conducted to assess whether the TyG index could predict the development of the NAFLD. The exclusion criteria were as follows: 1)、subjects who have already been diagnosed as DM, hypertension (HTN), dyslipidemia and MS or taking medicines for the above diseases. 2)、subjects who had NAFLD, viral liver diseases, autoimmune hepatic disease, or other chronic hepatic diseases based on the history and serology at study entry. 3)、subjects who were drinking alcohol greater than 140 g per week for males and 70 g per week for females. The subjects were reassessed after the annual physical health examinations. Eventually, a total of 4539 (2996 males and 1543 females) initially NAFLD-free subjects, were evaluated for the development of NAFLD.

### Clinical measurements and ultrasonography

All subjects accomplished a questionnaire, which included demographic characteristics such as alcohol consumption, smoking status and family history under the supervision of physicians who were well-trained in the medical examination center from the petrochemical hospital.

Height and weight of the subjects were measured with light indoor clothing and with no shoes. Waist circumference (WC) was calculated in standing subjects, midway between the lower edge of the costal arch and the top of the iliac crest. Body mass index (BMI, kg/m2), used as an index of body fat, was calculated as weight in kilograms divided by height in meters squared. Both WC and weight were measured in the morning before breakfast. The mean value of three blood pressure measurements was used to represent an individual’s blood pressure ultimately. Blood pressure was measured 3 consecutive times using the non-dominant arm with a 1-min interval between the measurements with the subjects in the seated position after 5 min of rest; and the automated device was used from Kyoto, Japan (Omron HEM-7052; Omron Corp.). Abdominal ultrasound (US) examination of the nonalcoholic fatty liver was performed and evaluated independently by experienced radiologists using a 3.5-MHz transducer (SoNoliNE Versa Plus, SIEMENS, Germany). In the supine position, subjects who participated in the US examination raised their right arm above the head. The diagnosis of NAFLD should have the presence of at least two of the following findings, according to the “Chinese Guideline on Diagnosis and Treatment of NAFLD” [16]: (1) diffuse increased echogenicity of liver compared with the kidney or spleen, (2) ultrasound beam attenuation, (3) poor visualization of intrahepatic structures.

After an overnight fasting for at least 10-h, a venous blood of three milliliter sample was obtained from each subject in the morning. All biochemical measurements, including total cholesterol (TC), TG, low density lipoprotein (LDL-C), high density lipoprotein cholesterol (HDL-C), creatinine (Cr), blood urea nitrogen (BUN), serum uric acid (SUA), FPG, aspartate aminotransferase (AST), ALT, γ-glutamyltransferase (γ-GGT), Apo-B and Apo-A1 were measured enzymatically using an Olympus AU640 auto-analyzer which was from Kobe, Japan. Also, all the laboratories involved resoundingly completed the standardization. The product of TyG was calculated using the following established formulas, TyG = Ln [TG (mg/ml) * FPG (mg/ml)/2] [17–19].

### Definitions

NAFLD was defined by the presence of abnormal findings in the US, and simultaneously ruled out the individuals with alcohol abuse (> 140 g per week for males and > 70 g per week for females) and competing etiologies for hepatic steatosis in accordance with the guidelines drafted by the Asia-Pacific Working Party [20]. The subjects who had three or more of the following abnormalities were diagnosed as MS: (I) blood pressure elevation, systolic pressure (SBP) ≥ 130 mmHg or diastolic pressure (DBP) ≥ 85 mmHg, or treatment of previously diagnosed HTN; (II) FPG elevation, defined as FPG ≥ 6.1 mmol/L, or previously diagnosed DM; (III) TG elevation, defined as triglycerides ≥1.7 mmol/L; (IV) reduced HDL-C, defined as HDL-C < 1.0 mmol/L; (V) WC ≥ 90 cm for Chinese males and ≥ 85 cm for Chinese females [21]. The calculation of estimated glomerular filtration rate (eGFR) was conducted through the MDRD equation modified by the Chinese [22].

### Statistical analysis

At baseline, four groups (quartile 1 (Q1) to quartile 4 (Q4)) were divided based on the TyG index. The classifications were as follows: 1)、for male: quartile 1(Q1) (≤ 8.09), quartile 2(Q2) (8.10–8.40), quartile 3(Q3) (8.41–8.76), and quartile 4(Q4) (≥ 8.77); and for female: quartile 1(Q1) (≤ 7.85), quartile 2(Q2) (7.86–8.15), quartile 3(Q3) (8.16–8.50), and quartile 4(Q4) (≥ 8.51). The baseline characteristics in each quartile were compared, and the cumulative incidence of NAFLD according to TyG index was also calculated.

Fundamental characteristics of the sample in the study were summarized by descriptive statistics. Data for continuous variables, which were expressed as median (IQR), were compared using the student’s t text, Mann-Whitney *U* test, Kruskal-Wallis *H* test or one way ANOVA depending on the normality; data for categorical variables, which were presented as percentages (%), were compared using Chi-square text. Cox proportional hazards regression was used to estimate hazard ratios (HR) for incident NAFLD for each TyG quartile. The hazard ratio and 95% confidence intervals (CI) were calculated for incident NAFLD. The model 1 was initially adjusted for gender and age, and then adjusted for indictors of MS (Model 2). Finally, the model 3 was further adjusted for other clinical factors. For linear trends of risk, the number of quartiles was used as a continuous variable and tested on each model. All statistics in the study were performed by the software SPSS 17.0 (SPSS Inc., Chicago, IL, USA). For a statistical inference, all *p* values are bilateral, and the difference had statistical significance if *p*-value was less than 0.05. A receiver operating characteristic (ROC) curve was constructed to assess the ability of the TyG index to predict the incident NAFLD, through comparing with the predictive value of TG, ALT and FPG.

## Results

### Baseline characteristics of the study population

A total of 4539 subjects (2996 males and 1543 females) were followed up in the study for 9 years, with the endpoint being the onset of NAFLD. Baseline clinical characteristics of the subjects in each TyG quartile were presented in Table 1. WC, age, BMI, SBP, FPG, DBP, UA, TC, LDL-C, Apo-B, TG, AST and ALT all tended towards increase with higher TyG index (*P* <  0.001), whereas eGFR and HDL-C decreased as the TyG increased (*P* <  0.001). Meanwhile, there was no significant difference among the quartiles in BUN, Cr and Apo-A1 (*P* > 0.05).

**Table 1:** Baseline characteristics of the subjects according to TyG quartiles

Variables	TyG quartiles				
	Quartile 1 (*n* = 1127)	Quartile 2 (*n* = 1146)	Quartile 3 (*n* = 1133)	Quartile 4 (*n* = 1133)	*p*
Gender (male/%)	751/66.6	754/65.8	748/66.0	743/65.6	0.96
Age (years)	38.0(32.0–45.0)	40.0(34.0–49.0)	43.0 (35.0–53.0)	47.0 (38.0–56.0)	< 0.001
BMI (kg/m^2^)	21.0 (19.6–23.0)	21.7 (20.0–23.7)	22.7 (21.0–24.4)	23.4 (21.8–25.0)	< 0.001
SBP (mmHg)	114.0 (106.0–123.0)	116.0 (108.0–126.0)	120.0 (111.0–129.0)	123.0 (114.0–132.0)	< 0.001
DBP (mmHg)	72.0 (67.0–78.0)	74.0 (68.0–80.0)	76.0 (70.0–83.0)	78.0 (73.0–84.0)	< 0.001
WC (cm)	73.0 (68.0–79.0)	75.0 (70.0–81.0)	78.0 (72.0–83.0)	80.0 (75.0–85.0)	< 0.001
TyG	7.79 (7.63–7.94)	8.17 (8.07–8.28)	8.49 (8.38–8.61)	8.97 (8.80–9.20)	< 0.001
BUN (μmol/l)	4.97 (4.24–5.79)	4.95 (4.25–5.77)	5.02 (4.18–5.87)	4.96 (4.17–5.76)	0.683
Cr (μmol/l)	72.0 (60.0–81.0)	73.0 (61.0–81.0)	73.0 (61.0–82.0)	71.0 (60.0–81.0)	0.353
FPG (mmol/l)	4.25 (4.00–4.52)	4.40 (4.12–4.67)	4.49 (4.21–4.84)	4.68 (4.33–5.11)	< 0.001
UA (μmol/l)	303.0 (247.0–350.0)	305.0 (251.8–357.3)	324.0 (262.0–384.0)	336.0 (276.0–390.0)	< 0.001
AST (U/l)	18.0 (16.0–22.0)	19.0 (16.0–22.0)	20.0 (17.0–23.0)	20.0 (17.0–24.0)	< 0.001
ALT (U/l)	18.0 (14.0–25.0)	20.0 (15.0–27.0)	22.0 (16.0–32.0)	24.0 (18.0–35.0)	< 0.001
y-GGT (U/l)	14.0 (11.0–19.0)	16.0 (11.0–21.0)	18.0 (13.0–27.0)	21.0 (15.0–35.0)	< 0.001
TC (mmol/l)	4.28 (3.78–4.82)	4.55 (4.04–5.09)	4.74 (4.20–5.33)	5.12 (4.54–5.77)	< 0.001
TG (mmol/l)	0.71 (0.60–0.82)	1.01 (0.88–1.14)	1.35 (1.16–1.54)	2.09 (1.71–2.65)	< 0.001
HDL-C (mmol/l)	1.38 (1.12–1.70)	1.31 (1.09–1.60)	1.24 (1.05–1.53)	1.24 (1.071.48)	< 0.001
LDL-C (mmol/l)	2.27 (1.90–2.68)	2.56 (2.06–3.03)	2.67 (2.24–3.20)	2.90 (2.36–3.44)	< 0.001
Apo-A1(g/l)	1.33 (1.17–1.50)	1.33 (1.16–1.50)	1.31 (1.13–1.50)	1.30 (1.12–1.53)	0.228
Apo-B(g/l)	0.77 (0.64–0.90)	0.85 (0.72–0.98)	0.93 (0.80–1.08)	1.04 (0.88–1.22)	< 0.001
eGFR (ml/(min·1.73 m^2^))	112.3 (99.9–127.7)	109.7 (99.9–123.4)	108.5 (96.6–123.0)	107.0 (96.0–123.4)	< 0.001

### Relationship between TyG index and incident NAFLD

**Fig. 1:**
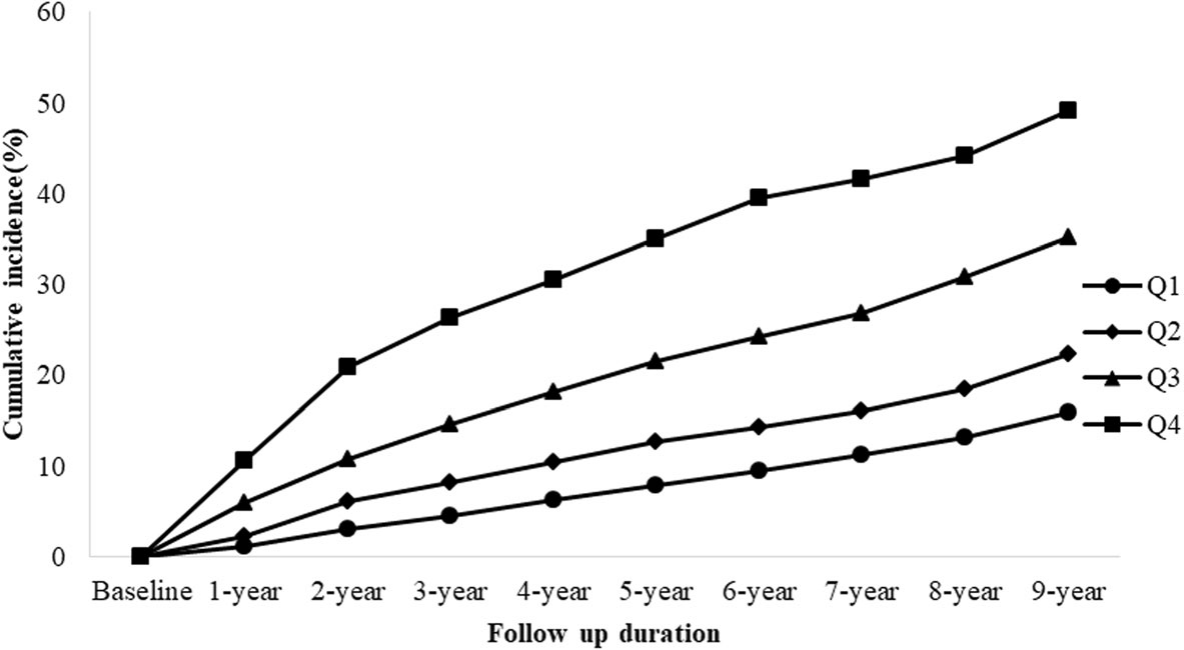
The association between baseline TyG index and the cumulative incidence of NAFLD

According to the baseline TyG index, the subjects were stratified into quartiles. During a total of 4539 person-years of follow-up, 1390 subjects including 1109 males and 281 females developed NAFLD, corresponding to 37.0 and 18.2% cumulative incidence of NAFLD in male and female, respectively (Table 2). In addition, Table 2 also demonstrated that the baseline BMI, gender, SBP, WC, DBP, γ-GGT, AST, TyG, ALT, BUN, Cr, UA, eGFR and lipids were all significantly different between two groups. Meanwhile, Fig. 1 showed that the baseline TyG quartiles predicted the incidence of NAFLD in a positive manner, and the overall 9-year cumulative incidence of NAFLD was 30.6%, ranging from 15.9% in Q1 to 22.3, 35.1 and 49.2% in Q2, Q3 and Q4, respectively (*P* for trend < 0.001). Above results demonstrated those with higher baseline TyG levels were more likely to develop NAFLD than those with lower levels.

**Table 2:** Baseline characteristics of the subjects according to follow-up outcomes

Variables	Subjects developed NAFLD (*n* = 1390)	Subjects did not develop NAFLD (*n* = 3149)	*P*
Gender (male/female, *n*)	1109/281	1887/1262	< 0.001
Age (years)	41.0(34.0–51.0)	41.0 (35.0–51.0)	0.226
BMI (kg/m^2^)	23.9 (22.2–25.3)	21.5 (19.9–23.3)	< 0.001
SBP (mmHg)	122.0 (114.0–131.0)	116.0 (107.0–126.0)	< 0.001
DBP (mmHg)	78.0 (72.0–84.0)	74.0 (68.0–80.0)	< 0.001
WC (cm)	82.0 (77.0–87.0)	74.0 (69.0–80.0)	< 0.001
TyG	8.58 (8.25–8.91)	8.21 (7.91–8.54)	< 0.001
BUN (μmol/l)	5.03 (4.23–5.86)	4.95 (4.19–5.75)	0.029
Cr (μmol/l)	75.0 (65.0–83.0)	70.0 (59.0–80.0)	< 0.001
FPG (mmol/l)	4.47 (4.15–4.82)	4.43 (4.14–4.77)	0.114
UA (μmol/l)	347.0 (294.0–401.0)	301.0 (245.0–356.0)	< 0.001
AST (U/l)	20.0 (17.0–24.0)	19.0 (16.0–22.0)	< 0.001
ALT (U/l)	26.0 (19.0–37.0)	19.0 (14.0–26.0)	< 0.001
y-GGT (U/l)	21.0 (15.0–33.0)	15.0 (11.0–22.0)	< 0.001
TC (mmol/l)	4.77 (4.17–5.41)	4.61 (4.03–5.24)	< 0.001
TG (mmol/l)	1.48 (1.10–2.04)	1.03 (0.79–1.39)	< 0.001
HDL-C (mmol/l)	1.20 (1.05–1.43)	1.33 (1.10–1.64)	< 0.001
LDL-C (mmol/l)	2.73 (2.26–3.26)	2.52 (2.04–3.05)	< 0.001
Apo-A1 (g/l)	1.26 (1.11–1.45)	1.35 (1.16–1.53)	< 0.001
Apo-B (g/l)	0.95 (0.80–1.14)	0.86 (0.72–1.03)	< 0.001
eGFR (ml/(min·1.73 m^2^))	108.1 (96.5–121.9)	110.8 (98.4–125.8)	< 0.001

### The TyG index and the risk of NAFLD

**Fig. 2:**
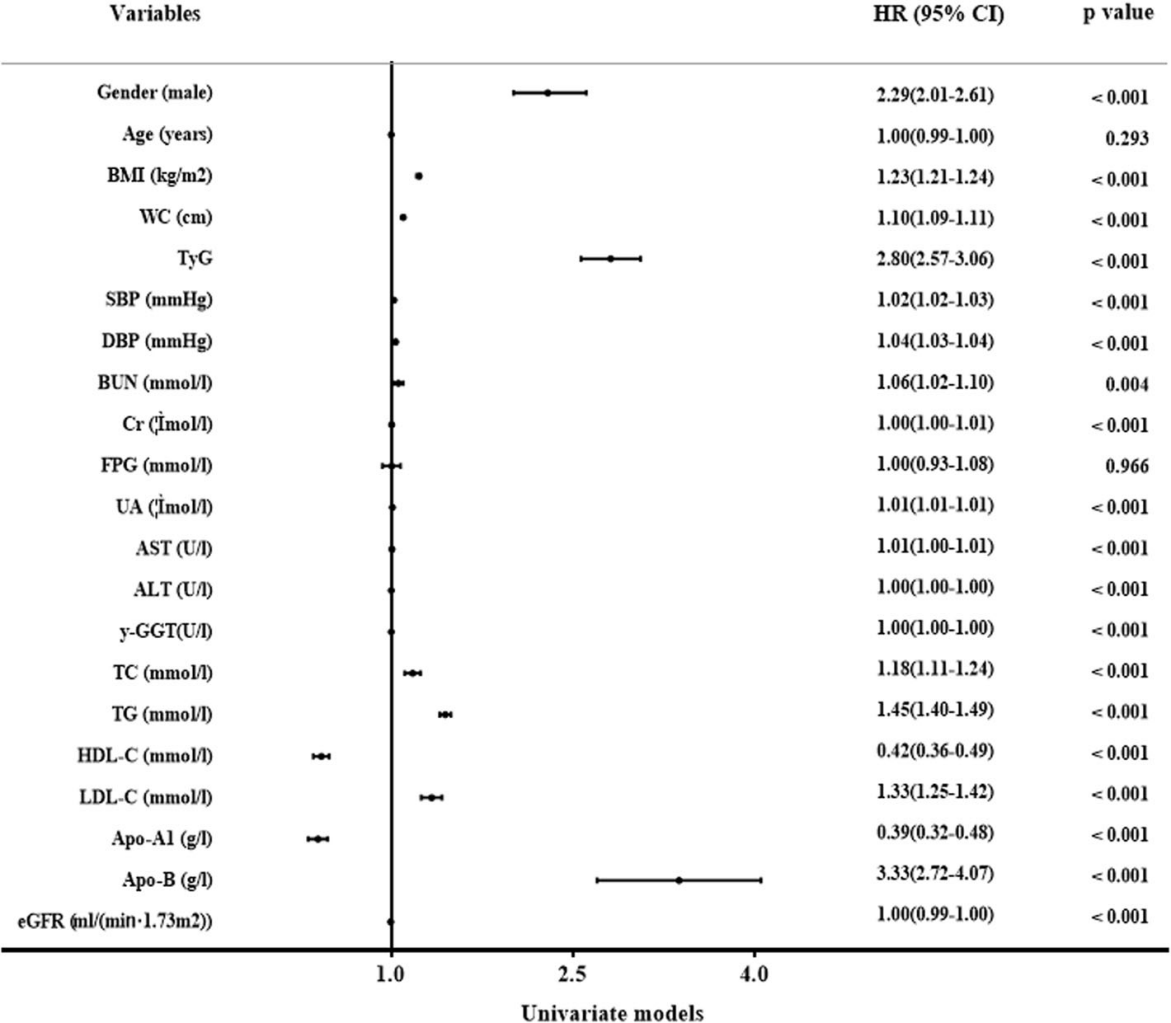
The univariate Cox hazard models of development of NAFLD during 9-year follow-up

**Fig. 3:**
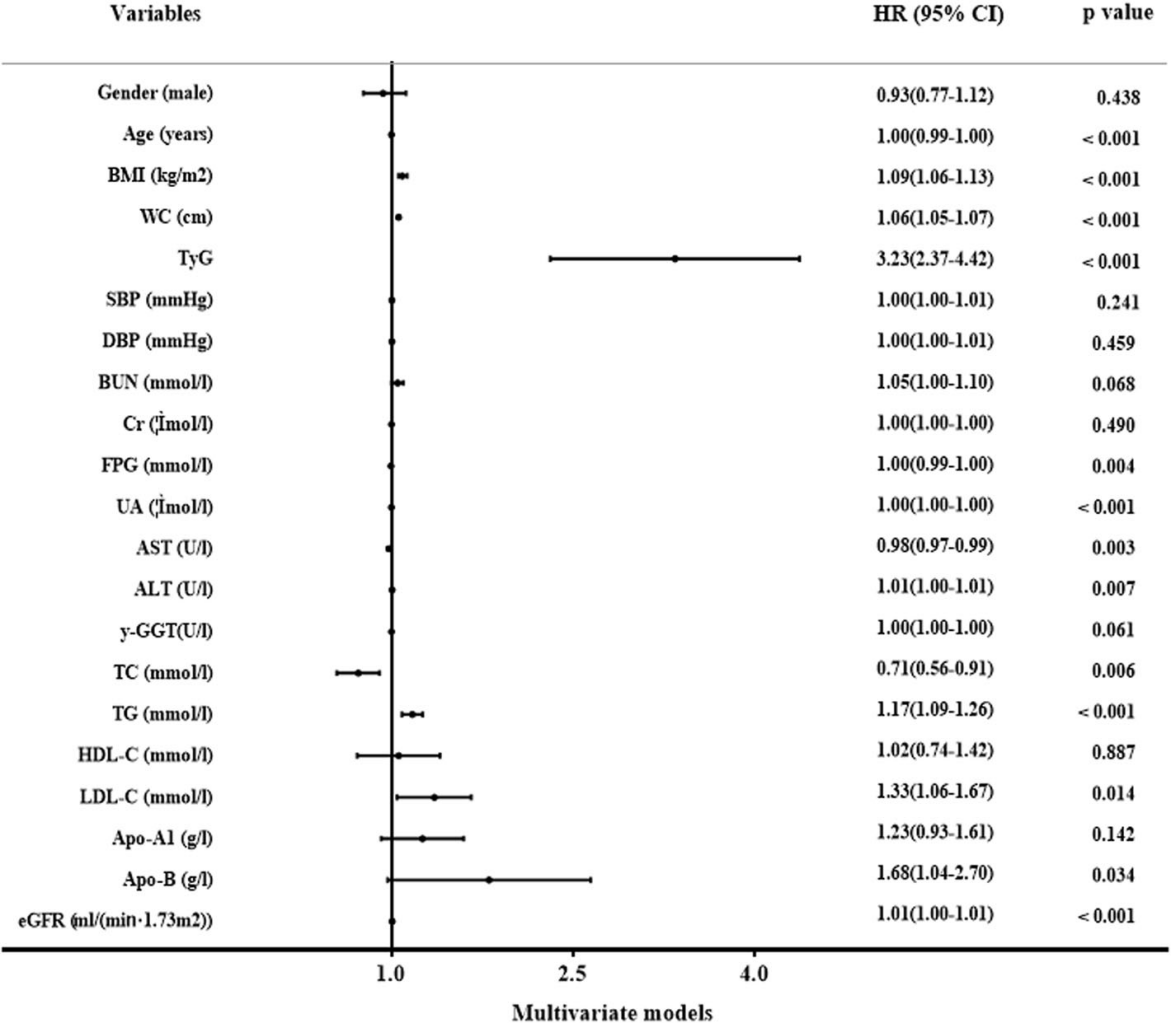
The multivariate Cox hazard models of development of NAFLD during 9-year follow-up

**Fig. 4:**
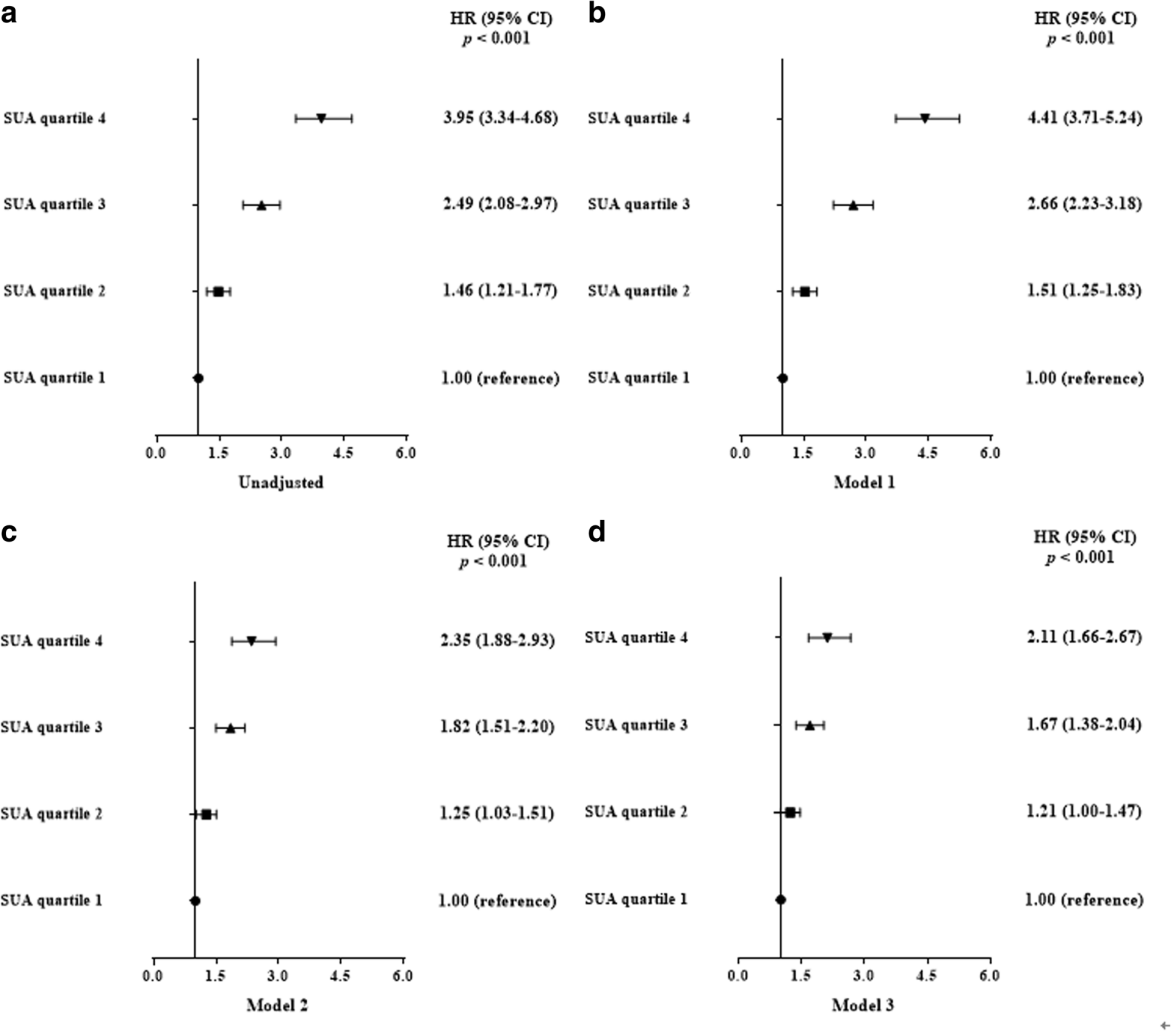
Risk of development NAFLD according to baseline TyG quartiles in unadjusted and adjusted models. Model 1: Adjusted for age and gender; Model 2: Adjusted for indictors of MS including age, gender, WC, SBP, DBP, FPG, HDL-C and TG; Model 3: Adjusted for age, gender, WC, BMI, SBP, DBP, TC, TG, HDL-C, LDL-C, Apo-A1, Apo-B, FPG, BUN, Cr, AST, ALT, y-GGT, UA and eGFR

We performed the analysis of TyG level and incident NAFLD using Cox proportional hazards regression models. It showed that gender, BMI, WC, TyG, SBP, DBP, γ-GGT, AST, ALT, BUN, Cr, UA, TC, HDL-C, TG, Apo-A1, LDL-C, eGFR and Apo-B were independent factors related with incident NAFLD in univariate models, while only Age, BMI, WC, AST, ALT, FPG, UA, TC, TG, eGFR, LDL-C, TyG and Apo-B were correlated with incident NAFLD in multivariate models (Figs. 2, and 3). Hazard ratio for incident NAFLD was also analyzed in each TyG quartile, with the first quartile serving as the reference group. The HR (95% CI) for subjects in Q2, Q3 and Q4 were 1.46(1.21–1.77), 2.49(2.08–2.97), and 3.95(3.34–4.68) (*P* for trend < 0.001) respectively, compared to the first quartile. Same conclusions were drew between TyG index and incident NAFLD even after adjusting for age and gender, or the indictors of MS or the clinical factors including BMI, WC, gender, SBP, age, DBP, TC, TG, Apo-A1, Apo-B, LDL-C, FPG, BUN, Cr, HDL-C, AST, ALT, UA, y-GGT and eGFR (Fig. 4). These above findings indicated TyG index was significantly related to an increased risk of subsequent incident NAFLD.

### Performance of ALT, TyG and its components for diagnosing subjects with NAFLD

**Fig. 5:**
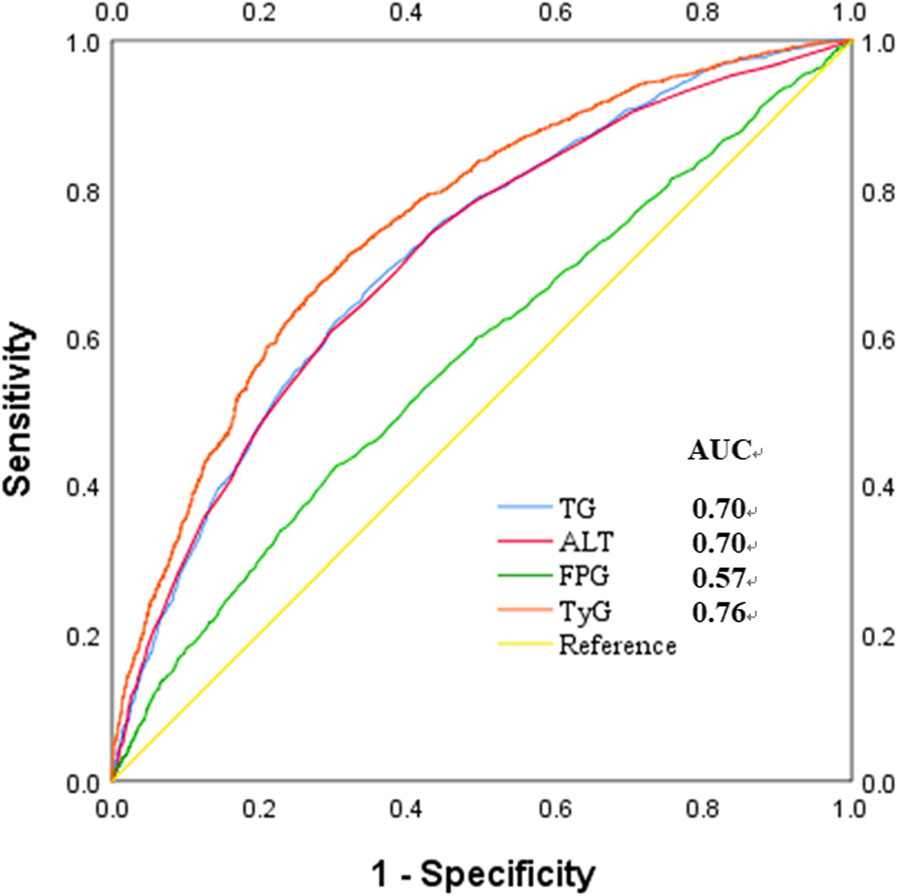
Receiver operative characteristic (ROC) curves and corresponding areas under the curve (AUC) for NAFLD. The AUC of TG, ALT, FPG and TyG were 0.70 (95% CI 0.69–0.72), 0.70 (95% CI 0.69–0.72), 0.57 (95% CI 0.55–0.59) and 0.76 (95% CI 0.74–0.77), respectively. *p* values for the difference between TyG and other two AUCs were <  0.0001

We further conducted a ROC curve analysis to assess the diagnostic value of ALT, TyG and its components. The area under the ROC (AUC) curve to analyze the ability of the baseline TyG to predict the development of NAFLD was 0.76 (95% CI 0.74–0.77), which was larger than that of TG (0.70 (95% CI 0.69–0.72), *P* for difference <  0.0001), ALT (0.70 (95% CI 0.69–0.72), *P* for difference <  0.0001), and FPG (0.57 (95% CI 0.55–0.59), *P* for difference <  0.0001). Based on the ROC plot, the optimal predictive cut-off of TyG for incident NAFLD was 8.52, with a sensitivity of 67.3% and a specificity of 71.9% (Fig. 5).

## Discussion

Currently, NAFLD has become one of the major causes of chronic liver disease worldwide [23]. Hence, taking account into the severity of the disease, the identification of an effective marker to predict the NAFLD is of great importance. This primary goal was to identify an index for predicting the incident NAFLD in a large healthy Chinese population. Over the 9-year period, about 30.6% of the subjects developed NAFLD. Our results indicate that the subjects with a high level of TyG index had a higher likelihood of suffering from NAFLD during the follow-up period. Cox regression also showed subjects who had a larger TyG index at baseline would significantly lead to a higher risk of incident NAFLD after adjusting for other risk factors. According to the ROC analysis, the TyG index was superior to its components and ALT in diagnosing NAFLD. These observations demonstrate that TyG index may be the effective predictor for developing NAFLD and the NAFLD risk increases along with the higher baseline TyG index.

Nowadays, several reports show that the TyG index is a biomarker for screening simple steatosis and NAFLD [24–26]; however, these studies are cross-sectional reports. The population-based findings in this study consolidate the relationship between TyG and NAFLD, and we further conclude that TyG is a longitudinal indicator of the later NAFLD, that is, TyG can predict the risk of NAFLD in the next few years. Also, we confirm that IR can lead to the occurrence of NAFLD in terms of epidemiology. In addition, increasing researches [1, 27] suggest that ALT value may accurately predict the NAFLD. In our study, ALT, which has become the primary screening tool for detecting acute liver injury [28, 29], was also associated with NAFLD; however, the AUC of the TyG index in diagnosing NAFLD was larger than that of ALT, FPG and TG in the ROC analysis. These indicated that TyG index was more effective for predicting the incident NAFLD, compared with the components of the TyG and ALT.

Though the mechanisms for NAFLD pathogenesis are still unclear, “two-hit hypothesis”, which has been obsolete recently, was widespread in past years. IR, as the main factor in the “first-hit”, induced the formation of reactive oxygen species (ROS) and dysfunction of mitochondria and constituted the initiating factor in NAFLD [30, 31]. The current evidence [32] suggests a “multiple-hit” hypothesis including IR, hormones secreted from the adipose tissue, nutritional factors, gut microbiota and genetic and epigenetic factors. From “two-hit” to “multiple-hit” hypothesis, IR plays an important role in the NAFLD throughout. Moreover, the severity of IR was also positively related to the progress and prognosis of NAFLD according to a recent study [33]. Therefore, the TyG index, which could identify the presence and severity of IR, may predict the subsequent occurrence of NAFLD in later life.

The association between TyG index and the incident diabetes mellitus has been found in a previous epidemiological study [34]. The results of our study showed the TyG index could predict the subsequent occurrence of NAFLD in later life. Hence, regular monitoring of the TyG index may benefit to prevent the occurrence of NAFLD in China.

The advantages of the study were as follows. First, our longitudinal study provided significant epidemiological evidence for the relation between TyG index and incident NAFLD in Ningbo, China. Moreover, the main strength was the large sample size and the prospective nature. However, some limitations also exist in our study. First, NAFLD was diagnosed only by ultrasonography, which cannot detect fatty infiltration < 10% [35]. This may lead to the underestimation of the true relationship between TyG index and incident NAFLD. Semi-quantitative ultrasonography may be more reliable for screening and understanding the pathogenesis of the NAFLD in future research [36]. Second, liver biopsy, which is the gold standard for the diagnosis of NAFLD, was not used because of the invasion. Third, we did not record the information on physical activity, which may affect the development of NAFLD. However, in this study, the duration and intensity of our subjects’ physical activity was almost similar every day, we did not think it would affect the results. Moreover, the information on nutritional habits or energy intake in this study was also not collected because we could not quantify above information every day. In another way, we used other additional covariates indirectly related to dietary, such as BMI or cholesterol for adjusting for this possible confounding factor. In addition, fasting insulin or the anti-inflammatory was also not obtained due to the lack of relevant devices and sufficient fund. Finally, the subjects of our study were the employees in a large-scale state-owned plant, and the conclusions may be different from the common population. A larger and multicenter study would be performed in future research.

## Conclusions

In summary, this population-based study indicated the TyG index could independently predict the incident NAFLD after adjustment for variables. Therefore, it may be suitable as a diagnostic criterion for NAFLD, and we may develop the strategy of NAFLD prevention based on the TyG index.

## Abbreviations

ALT: Alanine aminotransferase
AST: Aspartate aminotransferase
BMI: Body mass index
BUN: Blood urea nitrogen
Cr: Creatinine
CVD: Cardiovascular disease
DBP: Diastolic blood pressure
eGFR: Estimated glomerular filtration rate
FPG: Fasting plasma glucose
HDL-C: High density lipoprotein cholesterol
HTN: Hypertension
IR: Insulin resistance
LDL-C: Low density lipoprotein
NAFLD: Nonalcoholic fatty liver disease
SBP: Systolic blood pressure
SUA: Serum uric acid
TC: Total cholesterol
TG: Triglyceride
TyG: Triglyceride and glucose
WC: Waist circumference
γ-GGT: γ-glutamyltransferase

## References

[1] Ofliver EAF (2016). EASL–EASD–EASO clinical practice guidelines for the management of non-alcoholic fatty liver disease. J Hepatol.

[2] Loomba R, Sanyal AJ (2013). The global NAFLD epidemic. Nat Rev Gastroenterol Hepatol.

[3] López-Velázquez JA, Silva-Vidal KV, Ponciano-Rodríguez G (2014). The prevalence of nonalcoholic fatty liver disease in the Americas. Ann Hepatol.

[4] Li Z, Xue J, Chen P (2014). Prevalence of nonalcoholic fatty liver disease in mainland of China: a meta-analysis of published studies. J Gastroenterol Hepatol.

[5] Lazo M, Bilal U, Perez-Escamilla R (2015). Epidemiology of NAFLD and type 2 diabetes: health disparities among persons of Hispanic origin. Curr Diab Rep.

[6] Katsiki N, Mikhailidis DP, Mantzoros CS (2016). Non-alcoholic fatty liver disease and dyslipidemia: an update. Metabolism.

[7] Liu H, Lu HY (2014). Nonalcoholic fatty liver disease and cardiovascular disease. World J Gastroenterol.

[8] Lonardo A, Bellentani S, Non-alcoholic Fatty Liver Disease Study Group (2015). Epidemiological modifiers of non-alcoholic fatty liver disease: Focus on high-risk groups. Dig Liver Dis.

[9] Loria P, Marchesini G, Nascimbeni F (2014). Cardiovascular risk, lipidemic phenotype and steatosis. A comparative analysis of cirrhotic and non-cirrhotic liver disease due to varying etiology. Atherosclerosis.

[10] Younossi Zobair M., Blissett Deirdre, Blissett Robert, Henry Linda, Stepanova Maria, Younossi Youssef, Racila Andrei, Hunt Sharon, Beckerman Rachel (2016). The economic and clinical burden of nonalcoholic fatty liver disease in the United States and Europe. Hepatology.

[11] Lonardo A, Ballestri S, Marchesini G (2015). Nonalcoholic fatty liver disease: a precursor of the metabolic syndrome. Dig Liver Dis.

[12] Ballestri S, Zona S, Targher G (2016). Nonalcoholic fatty liver disease is associated with an almost twofold increased risk of incident type 2 diabetes and metabolic syndrome. Evidence from a systematic review and meta-analysis. J Gastroenterol Hepatol.

[13] Asrih M, Jornayvaz FR (2015). Metabolic syndrome and nonalcoholic fatty liver disease: is insulin resistance the link?. Mol Cell Endocrinol.

[14] Bugianesi E, Zannoni C, Vanni E (2004). Non-alcoholic fatty liver and insulin resistance: a cause-effect relationship?. Dig Liver Dis.

[15] Kim Bongyoung, Choi Hyun Young, Kim Wonhee, Ahn Chiwon, Lee Juncheol, Kim Jae Guk, Kim Jihoon, Shin Hyungoo, Yu Jae Myung, Moon Shinje (2018). The cut-off values of surrogate measures for insulin resistance in the Korean population according to the Korean Genome and Epidemiology Study (KOGES). PLOS ONE.

[16] Fan JG (2007). An introduction of strategies for the management of nonalcoholic fatty liver disease (NAFLD) recommended by Asia Pacific working party on NAFLD. Zhong hua Gan Zang Bing Za Zhi (Chinese).

[17] Simental-Mendía LE, Rodriguez-Morán M, Guerrero-Romero F (2008). The product of fasting glucose and triglycerides as surrogate for identifying insulin resistance in apparently healthy subjects. Metab Syndr Relat Disord.

[18] Guerrero-Romero F, Simental-Mendía LE, Rodriguez-Morán M (2010). The product of triglycerides and glucosea simple measure of insulin sensitivity. Comparison with the euglycemic-hyperinsulinemic clamp. J Clin Endocrinol Metab.

[19] Matthews DR, Hosker JP, Rudenski AS (1985). Homeostasis model assessment: insulin resistance and beta-cell function from fasting plasma glucose and insulin concentrations in man. Diabetologia.

[20] Farrell GC, Chitturi S, Lau GK (2007). Asia-Pacific working party on N. guidelines for the assessment and management of non-alcoholic fatty liver disease in the Asia-Pacific region: executive summary. J Gastroenterol Hepatol.

[21] Joint Commission on Revisions of Chinese Guideline for the Management of Dyslipidemia in Adults (2016). 2016 (in Chinese) Chinese guideline for the management of dyslipidemia in adults. Chin J Cardiol.

[22] Xun L, Cheng W, Hua T (2010). Assessing glomerular filtration rate (GFR) in elderly Chinese patients with chronic kidney disease (CKD): a comparison of various predictive equations. Arch Gerontol Geriatr.

[23] Younossi ZM, Koenig AB, Abdelatif D (2016). Global epidemiology of nonalcoholic fatty liver disease-meta-analytic assessment of prevalence, incidence, and outcomes. Hepatology.

[24] Simental-Mendía LE, Simental-Mendía E, Rodríguez-Hernández H (2016). The product of triglycerides and glucose as biomarker for screening simple steatosis and NASH in asymptomatic women. Ann Hepatol.

[25] Zhang S, Du T, Li M (2017). Triglyceride glucose-body mass index is effective in identifying nonalcoholic fatty liver disease in nonobese subjects. Medicine (Baltimore).

[26] Zhang S, Du T, Zhang J (2017). The triglyceride and glucose index (TyG) is an effective biomarker to identify nonalcoholic fatty liver disease. Lipids Health Dis.

[27] Pratt DS, Kaplan MM (2000). Evaluation of abnormal liver-enzyme results in asymptomatic patients. N Engl J Med.

[28] Senior JR (2012). Alanine aminotransferase: a clinical and regulatory tool for detecting liver injury-past, present, and future. Clin Pharmacol Ther.

[29] McGill MR (2016). The past and present of serum aminotransferases and the future of liver injury biomarkers. EXCLI J.

[30] Day CP, OF J (1998). Steatohepatitis: a tale of two “hits”?. Gastroenterology.

[31] Gaggini M, Morelli M (2013). Non-alcoholic fatty liver disease (NAFLD) and its connection with insulin resistance, dyslipidemia, atherosclerosis and coronary heart disease. Nutrients.

[32] Buzzetti E, Pinzani M, Tsochatzis EA (2016). The multiple-hit pathogenesis of non-alcoholic fatty liver disease (NAFLD). Metabolism.

[33] Samuel Varman T., Liu Zhen-Xiang, Qu Xianqin, Elder Benjamin D., Bilz Stefan, Befroy Douglas, Romanelli Anthony J., Shulman Gerald I. (2004). Mechanism of Hepatic Insulin Resistance in Non-alcoholic Fatty Liver Disease. Journal of Biological Chemistry.

[34] Navarro-González D, Sánchez-Íñigo L, Pastrana-Delgado J (2016). Triglyceride-glucose index (TyG index) in comparison with fasting plasma glucose improved diabetes prediction in patients with normal fasting glucose: the vascular-metabolic CUN cohort. Prev Med.

[35] Ballestri S, Nascimbeni F, Baldelli E (2017). Ultrasonographic fatty liver indicator detects mild steatosis and correlates with metabolic/histological parameters in various liver diseases. Metabolism.

[36] Ballestri S, Romagnoli D, Nascimbeni F (2015). Role of ultrasound in the diagnosis and treatment of nonalcoholic fatty liver disease and its complications. Expert Rev Gastroenterol Hepatol.

